# Discounting of reward sequences: a test of competing formal models of hyperbolic discounting

**DOI:** 10.3389/fpsyg.2014.00178

**Published:** 2014-03-06

**Authors:** Noah Zarr, William H. Alexander, Joshua W. Brown

**Affiliations:** ^1^Deparment of Psychological and Brain Sciences, Indiana UniversityBloomington, IN, USA; ^2^Department of Experimental Psychology, Ghent UniversityGhent, Belgium

**Keywords:** discounting, hyperbolic discounting, exponential discounting, model fitting, Parallel model, temporal difference learning, recursive model, behavioral research

## Abstract

Humans are known to discount future rewards hyperbolically in time. Nevertheless, a formal recursive model of hyperbolic discounting has been elusive until recently, with the introduction of the hyperbolically discounted temporal difference (HDTD) model. Prior to that, models of learning (especially reinforcement learning) have relied on exponential discounting, which generally provides poorer fits to behavioral data. Recently, it has been shown that hyperbolic discounting can also be approximated by a summed distribution of exponentially discounted values, instantiated in the μAgents model. The HDTD model and the μAgents model differ in one key respect, namely how they treat sequences of rewards. The μAgents model is a particular implementation of a Parallel discounting model, which values sequences based on the summed value of the individual rewards whereas the HDTD model contains a non-linear interaction. To discriminate among these models, we observed how subjects discounted a sequence of three rewards, and then we tested how well each candidate model fit the subject data. The results show that the Parallel model generally provides a better fit to the human data.

## Introduction

In the past two decades, models of reinforcement learning (RL) have revolutionized our understanding of the neural bases of reward processing and decision-making. Early single-unit neurophysiology studies in monkey identified dopamine (DA) neurons in primate ventral tegmental area (VTA) whose activity over the course of learning developed in the same manner as a reward prediction error in temporal difference learning (for a review, see Schultz, 1998). Since then, studies in which key aspects of the RL framework have been applied to the interpretation of neural activity have proliferated. Theoretical work has attempted to link neuromodulatory systems in addition to DA to key parameters in temporal difference (TD) learning (Daw et al., 2002; Doya, 2002; Krichmar, 2008; Smith et al., 2013). At the same time, empirical work has identified neural signatures corresponding to quantities that are presumed to be used in RL learning, including reward value (O'Doherty et al., 2004), delay until a reward is received (Kable and Glimcher, 2007), and the probability of failing to receive a reward (Brown and Braver, 2005).

Despite its ubiquity in modern neuroscience research, key questions remain to be resolved if the RL framework is to continue to be a useful paradigm for advancing our understanding of the brain (Dayan and Niv, 2008). Significant among these is the question of how the present value of future rewards is calculated. Given a choice between a reward now and the same reward later, humans and animals generally prefer the immediate reward. Temporal discounting refers to the phenomenon whereby the value of a rewarding state of affairs is diminished by the delay until that state of affairs obtains. A common assumption in RL models is that future rewards are discounted exponentially:

(1) $$V=Re^{-kT}$$

where *V* is the value of a delayed reward, *R* is the magnitude of reward, *k* is a discount parameter, and *T* is the delay to the reward. Exponential discounting is ubiquitous in RL models chiefly due to computational convenience stemming from its ability to be defined recursively:

(2) $$V_{t+1}=V_{t}e^{-k}$$

In this model, the value at each timestep is multiplied by the same amount, namely *e*^−*k*^. Although the Exponential model of discounting is computationally convenient, the preponderance of behavioral evidence reveals that animals (e.g., Ainslie and Herrnstein, 1981; Green and Estle, 2003) and humans (e.g., Green et al., 1994; Kirby and Herrnstein, 1995) discount future rewards hyperbolically. Simple hyperbolic discounting can be defined as:

(3) $$V=\frac{R}{1+kT}$$

The effect of this formulation is that value is now inversely proportional to the length of the delay. This is in contrast to the Exponential model in which the value is reduced by a fixed percentage at each timestep. Within the brain, it has been observed that the activity of DA neurons in VTA is more consistent with hyperbolic discounting (Schultz, 2010). Furthermore, it has been suggested that the preference reversals (inconsistency of intertemporal choice) characteristic of hyperbolic discounting are due to electrical coupling in DA neurons via gap junctions (Takahashi, 2007). Although evidence for hyperbolic discounting predominates at the behavioral and neural levels, standard RL formulations tend not to incorporate hyperbolic discounting, in part because of its previously presumed lack of a recursive definition (Dayan and Niv, 2008).

Recently, two RL models have been proposed which are able to exhibit hyperbolic discounting. In Alexander and Brown (2010), the authors show how hyperbolic discounting can in fact be defined recursively as

(4) $$V_{t}=\frac{R_{t+1}+V_{t+1}}{1+\frac{kV_{t+1}}{\bar{r}}}$$

where *r* is a scaling factor equal to the reward expected for a given trial. This scaling factor is needed because discounting is a function of the discount factor and *V*_*t*_. Therefore as the magnitude of reward increases, driving up *V*_*t*_, the rate of discounting would increase inappropriately. In contrast to the non-recursive definition of hyperbolic discounting [Equation (3)], the time to a reward is not represented explicitly in the hyperbolically discounted temporal difference (HDTD) model. Instead, the time to a reward is implicitly represented by the degree to which the value of that reward is discounted. Because value at a given time is determined based on the value at the next timestep, this model predicts that the effective discounted value of a reward will be increased by the presence of additional rewards. As a result, the values of rewards in a sequence combine superadditively. This is the key difference in the predictions generated by the HDTD model compared to the more standard Parallel model, discussed below.

The HDTD model can also be formulated with an additional parameter,

(5) $$V_{t}=\frac{R_{t+1}+V_{t+1}}{1+\frac{kV_{t+1}}{{\bar{r}}^{\sigma }}}$$

Here σ allows discounting to vary non-linearly with reward magnitude. In all other respects, this model is identical to the previously discussed HDTD model described by Equation (4).

A second approach to recursive temporal discounting, the μAgents model (Kurth-Nelson and Redish, 2009), shows that hyperbolic discounting can be achieved by averaging over multiple exponential discounting functions. In the μAgents model, each exponential discounting function has a distinct discount parameter, and the discount parameters for all exponential discounting functions are distributed in some fashion. The distribution used influences the effective hyperbolic discount parameter. Because each exponential function is itself expressible recursively, the μAgents model shares with the HDTD model the advantage of being usable in TD learning. Sozou (1998) demonstrated that for certain distributions of exponential discount parameters, models such as μAgents are formally equivalent to the simple hyperbolic model [Equation (3)].

Although both the HDTD and μAgents models have been demonstrated to be mathematically equivalent to the simple model of discounting [Equation (3)] in the special case of a single future reward, they suggest different mechanisms by which hyperbolic discounting might arise. The μAgents model proposes that the value of future rewards is represented as the sum of a distribution of exponentially discounted values of a future reward, while the HDTD model suggests that value is represented as the future value of a reward scaled by the reward magnitude. These distinct mechanisms lead to a number of differing predictions.

One such example is explored in Kurth-Nelson and Redish (2010), in which they appear to demonstrate that the μAgents model, and not the HDTD model, is able to exhibit *precommitment*, the ability for humans and animals to ensure that a larger, later reward will be obtained by acting to preclude the possibility of selecting a smaller, more immediate reward (e.g., one can precommit to saving money over buying a new gadget by not going to the store). However, in unpublished simulations, we observed that a minor adjustment to the HDTD model would allow it to precommit. Specifically, in the published version of the HDTD model (Alexander and Brown, 2010), *r* represented the total amount of reward available during a single trial. If instead we specify that *r* represents the total remaining reward available on a trial, we observe precommitment behavior in the HDTD model. The question of which model can better account for discounting behavior therefore remains very much open.

In order to answer this question, we identified another means by which the models may be discriminated. Specifically, the two models differ in how they treat sequences of rewards. Because exponential functions are memoryless, the exponentially discounted value of a reward does not depend on other rewards nearby. Since the μAgents model relies on exponential discounting for its overall hyperbolic discounting, it also has this property. The value of a sequence at a given time is simply the sum of the individually discounted rewards at that time. In short, individual rewards in a sequence are discounted independently of one another.

This is equivalent to a previous proposal regarding discounting of multiple rewards, the Parallel hyperbolic model (Brunner and Gibbon, 1995). In this model, total value of a reward sequence is computed by summing over the values of future rewards, each of which has been separately discounted using the simple hyperbolic model.

(6) $$V=\sum_{n=1}^{N}\frac{R}{1+kT_{n}}$$

Here, *n* indexes rewards, *R* represents the magnitude of each reward, *k* is the discount parameter, and *T* represents the amount of time until the reward will be obtained.

Like the HDTD model, the Parallel model also has a two parameter version,

(7) $$V=\sum_{n=1}^{N}\frac{R}{{\left(1+kT_{n}\right)}^{\sigma }}$$

The σ parameter alters the shape of the hyperbola, resulting in a shallower curve for values less than 1. The addition of this parameter has been shown to provide a better fit to behavioral data (Myerson and Green, 1995).

Because μAgents is equivalent to the Parallel discounting model, we used the Parallel model as a computationally convenient stand-in for the μAgents model. The Parallel model has the advantage that it can generate the value of a delayed reward in one step without requiring the separate calculation of many exponentially delayed values. In addition, it has an easily interpretable discount parameter which is not the case for the μAgents model. In that model, the discount parameters are sampled from a distribution which may itself have multiple parameters. This would greatly complicate model fitting and require invoking additional assumptions. Since both models constitute linear combinations of sequential, hyperbolically discounted rewards, the Parallel model is an appropriate substitute for the μAgents model in this context. While the two models make identical predictions in terms of behavior, the μAgents model is readily interpretable as a collection of “micro-agents” all discounting in Parallel, perhaps subserved by individuated loops through the basal ganglia (Kurth-Nelson and Redish, 2009). Differences in neural implementation, however, are not directly relevant to the current project.

In contrast to the Parallel model, the HDTD model modulates reward value as a result of nearby rewards. Because value at a given time is determined based on the value at the next timestep, this model predicts that the effective discounted value of a future reward will be increased by the presence of additional rewards nearby in time. In other words, the values of rewards in a sequence combine superadditively.

We therefore aim to discriminate between these two primary models utilizing behavioral data from a task which determines how much subjects value various hypothetical sequences of monetary rewards. Because two-parameter models may offer a better fit to human discounting data, even when they are penalized for having the extra parameter (Peters et al., 2012), we also looked at two-parameter versions of the HDTD and Parallel models. Our design has the benefit of providing data on how subjects discount sequences of rewards, an issue which has implications extending beyond the HDTD vs. μAgents debate. This is especially true since many decisions have consequences that are spread out in time, yet most studies of discounting deal with only single rewards. We will consequently use the data generated in the experiment to test between a number of discounting models in addition to the four presented above.

First, we tested the standard Exponential model as a sort of baseline. Like the Parallel model, the Exponential model does not treat rewards differently based on the presence of nearby rewards. Therefore, to compute the value of a reward sequence at a given point the values of the individual rewards at that time are simply summed.

(8) $$V=\sum_{n=1}^{N}R^{-kT_{n}}$$

We also examined two models created and tested in rats by Brunner and Gibbon (1995). The first of these is the Mixed model, so named because the model treats rewards separately in some sense but also discounts the sequence as a holistic package. The value of the sequence is computed by discounting each reward hyperbolically from the time of the first reward. So the value of the package presented immediately is,

(9) $$V_{0}=\sum_{n=1}^{N}\frac{R}{1+\left(n-1\right)kT_{s}}$$

where *n* indexes the reward and *T*_*s*_ is the spacing between rewards. The package is then itself discounted hyperbolically,

(10) $$V=\frac{V_{0}}{1+kT_{f}}$$

where *T*_*f*_ is the delay before the first reward.

Finally, we tested the Serial model, also from Brunner and Gibbon (1995). In the Serial model, each reward is discounted based on its distance from the previous reward. The first reward is discounted according to the temporal distance between the current time and the first reward. The second reward is then discounted based on the distance between the first and second rewards, etc. For a sequence of three rewards value is therefore computed as,

(11) $$V=\frac{R+\frac{R+\frac{R}{1+kT_{s}}}{1+kT_{s}}}{1+kT_{f}}$$

where, again, *T*_*s*_ is the spacing between rewards and *T*_*f*_ is the delay before the first reward, i.e., the onset in the behavioral task.

## Materials and methods

### Participants

Twenty-five people (14 females) participated in this study. The average age was 22 years (*SD* = 3.97). Participants were recruited through advertisements posted on the university campus and nearly all participants were Indiana University students. The experiment took approximately 40 min–1 h and participants were compensated with $10. All subjects gave informed consent prior to their participation in the study.

### Task

**Figure 1:**
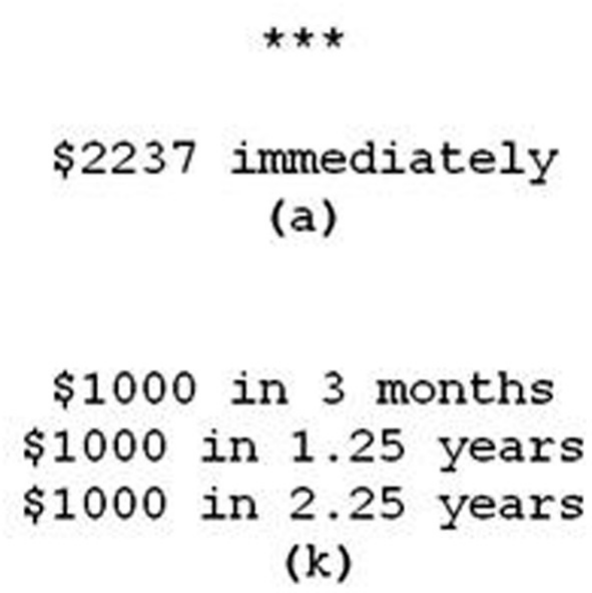
**An example display of a choice as seen by participants**.

The task consisted of a series of choices between an immediate monetary reward and a delayed monetary reward. Depending on condition, the delayed reward could be given all at once or as a sequence of rewards separated by some time intervals. All rewards were purely hypothetical. Because a primary goal of this experiment was to understand the effects of reward sequences on temporal discounting, in most cases the delayed reward consisted of three payments arranged in a series. In these cases each of the three payments was $1000. The other cases consisted of conditions in which a delayed reward of $3000 or $1000 was administered all at once. The payment schedule of the delayed reward was manipulated within subjects along two orthogonal dimensions. The first dimension was the time until the first reward in the series, which we called the onset. The onset could be 3, 6, 12, 24, 36, 48, or 84 months. The second dimension was the spacing between the reward sequences which could be 0, 12, or 60 months. The “0” spacing represents two conditions in which the delayed reward was given in one lump sum, either $3000 or $1000. The different sizes of the lump sum reward were included in order to examine how the rate of discounting is affected by differently sized rewards without the confound of a reward sequence vs. lump sum payment. Participants saw every combination of spacings and onsets. For example, one condition was an onset of 6 months and a spacing of 12 months. In this case, the delayed option would consist of $1000 in 6 months, $1000 in 18 months, and $1000 in 30 months. Time spans of less than a year were displayed in units of months whereas time spans of a year or more were displayed in terms of years, as shown in Figure 1.

For each spacing-onset pair, participants completed 14 consecutive trials. During each of these blocks of trials, the reward offered immediately was adjusted according to a staircase procedure. The goal of the procedure was to determine the immediate reward value such that participants were indifferent between the immediate and delayed options. In other words, we were attempting to determine the value each participant placed on each of the delayed reward schedules. Possible values ranged from $1 to $3000 since presumably participants would always take the delayed option rather than nothing and prefer the $3000 now as opposed to in the future.

The staircase procedure started at the midpoint of this interval plus a small amount of noise. Therefore, the first immediate option presented to the participants for each condition was around $1500. The interval of possible indifference points was then based on the participant's responses. The simplest staircase procedure would, on each trial, set one of the interval endpoints to the value which was offered immediately. For example, if the immediate option was $1500 and the participant chose the delayed option, in effect saying the immediate offer was too low, the interval of possible indifference points would be adjusted from 0–3000 to 1500–3000. However, if decision making is a noisy or stochastic process (e.g., Johnson and Busemeyer, 2005), this procedure would result in certain values being excluded prematurely. For instance the person's “true” (or mean) indifference point could be $1400 but due to noise in the decision making process she might chose the delayed option when offered $1500 immediately. Now no values lower than $1500 would ever appear again in the set of 14 trials for that condition and the true indifference point can never be reached. To address this issue, we modified the staircase procedure such that the new interval excludes 60% rather than 100% of the values between the old interval endpoint and the offered amount. So if a participant chose the delayed option when offered $1500 immediately, instead of moving the interval minimum from 0 to 1500, it would move to 900.

Over the course of 14 trials, the staircase procedure yielded a narrow range of possible indifference points for that particular condition (i.e., combination of onset and spacing). The indifference point was assumed to be the mean of this interval which was recorded for use in the model fitting before participants moved on to another 14-trial block in a different condition. Conditions were presented sequentially such that after completion of each condition the onset was increased to the next level. If the onset was already 84 months (the highest level), the spacing was increased to the next level and the onset was reset back to 0. This procedure yielded 7 indifference points per spacing, one for each onset. These points could then be used to form a discounting curve, indicating how the value of a reward sequence changes based on how far in the future it is. We then fit the models, which yield similar discounting curves, to the data-based curves and calculated the degree of fit.

The experiment was completed on PCs using E-Prime 2.0 (Psychology Software Tools, Pittsburgh, PA). On each trial, participants were presented with two options on the screen, one displayed above the other as in Figure 1. Which choice appeared on top was held constant within the 14 trials dedicated to a particular condition but varied randomly between conditions. While this was done to alleviate order effects, participants were of course free to look at the options in any order and this order may have been affected by the various amounts offered in addition to their spatial arrangement. The “a” key was always used to select the top option and the “k” key was always used to select the bottom option. Participants could not make a selection and advance to the next choice until a 4 s delay period elapsed. This was signified by the appearance of three asterisks at the top of the screen. This period was put in place to help ensure that participants were actually weighing the choices and not merely advancing as quickly as possible.

### Analysis

Each of the models was fit to each subject's data individually by varying the discount parameter and the sigma parameter where appropriate. After fitting, the Bayesian information criterion (BIC) was computed for each model (Schwarz, 1978; Priestley, 1981).

(12) $$\text{BIC}=n\cdot \ln\text{​}\left(\sigma_{e}^{2}\right)+p\cdot \text{ln}(n)$$

where *n* is the number of data points (28 per participant per model) and *p* is the number of free parameters (1 or 2 depending on the model).

Here σ^2^_*e*_ is the error variance, defined as

(13) $$\frac{1}{n}\sum_{i=1}^{n}{\left(m_{i}-b_{i}\right)}^{2}$$

where *m* is the model-derived indifference point and *b* is the observed behavioral indifference point. We then performed a matched-samples ANOVA and Bonferroni-corrected pairwise matched-samples *t*-tests, using the various models' BICs for a particular subject as the matched samples (cf. Milosavljevic et al., 2010).

## Results

**Figure 2:**
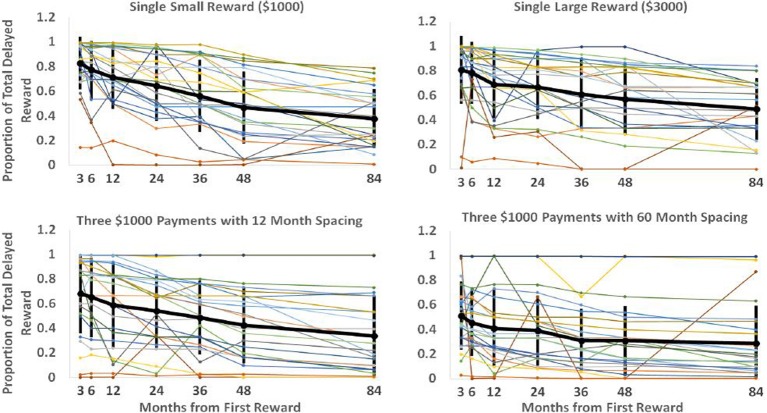
**Each plot displays each participant's discounting curve for a particular delayed reward schedule.** The vertical axis represents the proportion of total available reward subjects would have to be given immediately for them to be indifferent between the immediate and delayed rewards. The horizontal axis represents the delay before the first reward in the sequence would be received. The black line shows the mean responses with error bars showing the standard deviation of responses for that particular spacing and onset time.

Each “spacing” condition constitutes a particular reward schedule. By determining indifference points for these schedules with a varying interval until the first reward, we can see how the value of these schedules drops off as the time to reach them increases. These curves are displayed in Figure 2 below. Each of the plots represents a particular reward schedule, with the narrow lines representing the data generated by particular participants. The bold line indicates the mean, with error bars of one standard deviation. As observed in previous studies on discounting (e.g., Myerson and Green, 1995), it is readily apparent that the data are quite noisy, with some participants displaying non-monotonic value judgments as time to reward increases and others displaying little discounting. Nevertheless, we were able to use this data to discriminate between the models.

A BIC was calculated for each model for each participant. The average BIC for each model is displayed in Table 1 with the models listed from best to worst. “Parallel2” and “HDTD2” refer to the two-parameter versions of the respective models. In addition, we calculated how many subjects had data best fit by each particular model. Notably, the Exponential model offered the best fit for the plurality of subjects. Since every other model utilized hyperbolic rather than exponential discounting, this likely reflects the fact that the other models are more similar to each other than to the exponential model. If we consider that 78% of the time a hyperbolic model provided the best fit, our data are in accord with previous work on hyperbolic vs. exponential discounting in which data from some subjects is better fit by exponential discounting (Myerson and Green, 1995).

**Table 1:** **Model fitting results**.

**Model**	**Average BIC**	**BIC standard error**	**Subjects best fit by model**
Parallel2	321.87	6.19	5 (20%)
Mixed	322.37	6.16	3 (12%)
Parallel	324.61	6.11	2 (8%)
Exponential	325.85	5.91	7 (28%)
HDTD	327.02	5.77	3 (12%)
HDTD2	327.85	5.87	0 (0%)
Serial	333.76	6.08	5 (20%)

We used a One-Way repeated measures ANOVA to test whether certain models fit better than others, taking into account that each model was fit to each participant. Based on Mauchly's test of sphericity, our data violated the assumption of sphericity, *p* < 0.001. A Greenhouse-Geisser-corrected *F*-test with a Box index of sphericity of 0.271, and corrected degrees of freedom demonstrated that the goodness of fit between models was significantly different, *F*_(1.628, 19)_ = 4.689, *p* = 0.021.

We then completed *post-hoc* pairwise comparisons using matched-samples *t*-tests with a Bonferonni-corrected alpha level of *p* = 0.0024 and 24 degrees of freedom. The matched samples were the list of BICs generated by each model, one for each participant. Our results are summarized in Table 2 below.

**Table 2:** **Pairwise comparisons between models based on BIC scores**.

	**HDTD**	**Parallel**	**Exponential**	**HDTD2**	**Parallel2**	**Mixed**	**Serial**
HDTD							
Parallel	*t* = 3.90, *p* < 0.001^*^ (Parallel)						
Exponential	*t* = 0.64, *p* = 0.53	*t* = −0.67, *p* = 0.51					
HDTD2	*t* = −1.41, *p* = 0.17	*t* = −4.3, *p* < 0.001^*^ (Parallel)	*t* = −1.05, *p* = 0.31				
Parallel2	*t* = 3.75, *p* < 0.001^*^ (Parallel2)	*t* = 2.18, *p* = 0.04	*t* = 1.82, *p* = 0.08	*t* = 3.74, *p* = 0.001^*^ (Parallel2)			
Mixed	*t* = 4.31, *p* < 0.001^*^ (Mixed)	*t* = 3.34, *p* = 0.0028	*t* = 2.32, *p* = 0.029	*t* = 4.92, *p* < 0.001^*^ (Mixed)	*t* = −0.33, *p* = 0.74		
Serial	*t* = −1.50, *p* = 0.15	*t* = −2.18, *p* = 0.04	*t* = −1.69, *p* = 0.10	*t* = −1.34, *p* = 0.19	*t* = −2.71, *p* = 0.01	*t* = −2.88, *p* = 0.008	

An asterisk indicates significance. For significant comparisons, the winning model is in parentheses.

Since the Parallel2 model was most successful in accounting for participants' data, the best-fitting parameters for this model are of particular interest. For a number of reasons, however, they do not offer a straightforward interpretation. Recall that the Parallel2 model has a discount parameter *k*, and a parameter σ which raises the denominator to a power. The latter has been interpreted as a non-linear scaling parameter reflecting the relationship between subjective and objective reward magnitude (Myerson et al., 2001). However, because the *k* and σ parameters are correlated (i.e., an increase in either parameter results in increased discounting), the best-fit values obtained for each parameter are unreliable in that the data may be fit equally well by a range of values for *k* and σ. As a consequence of the correlation between parameters, the values obtained for each parameter from our model fit were highly variable; the distribution of discount parameters for this model had a mean of 7.05E+304, a median of 0.031, a range of 1.18E-08 to 8.81E+305 with a standard deviation of 2.39E+305. The best-fitting σ values had a mean of 1.92E+05, a median of 1.01, a range of 9.83 E-05 to 9.57E+05, and a standard deviation of 2.98E+05. The extreme values were likely the joint product of the interaction between the two parameters in combination with outlying subject data. As can be seen in Figure 2, some curves are quite flat and remain at very high or very low values. Two such curves are responsible for the maximum discount parameter of 8.81E+305 being the best fit for two participants. Such values (which likely reflect computational limits as *k* was driven upward) can provide a better fit to some curves due to the interaction with σ which is driven very low in such cases.

The solution of a very large discount factor with a very small σ value was not only found for the outlying data, however. For the more normal curves, the solutions generally fell into two clusters. One cluster consisted of discount parameters between 1 and 0.001 with σ values between 1 and 0.1. The other cluster featured very low discount parameters on the order of 10 - 08 and σ values on the order of 10 + 05. Naturally, such values do not readily afford psychological interpretation.

While σ may offer a significantly better fit to some data, it comes at the cost of making some optimal solutions unintelligible. The best-fitting discount parameters for the standard Parallel model, which lacks a σ parameter, are more manageable. They were distributed with a mean of 0.15, a median of 0.08, a range of 0.04–1.36, and a standard deviation of 0.27. These values are much less extreme and readily offer an interpretation of the discount parameter as corresponding to value placed on not having to wait for reward.

This experiment also allowed us to examine how different sizes of rewards influenced the discount rate. The magnitude effect refers to the finding that higher rewards are discounted less sharply than smaller rewards. This effect has been found in a variety of studies (e.g., Myerson and Green, 1995; Kirby, 1997; Johnson and Bickel, 2002), and would predict that the best-fitting discount parameters in the single $3000 reward case would be smaller on average than those found in the single $1000 reward condition. From there, it would be interesting to see whether sequences of three $1000 rewards are discounted more like the $1000 or $3000 lump sum. To investigate this, we looked at the average best-fitting discount rates for the Parallel model, despite it not being the best fitting model. We did this both because the Parallel model is one of the two key models we examined and also because the discount parameter has a more straightforward interpretation in the Parallel model as opposed to the Parallel2 and Mixed models which performed slightly better. In the case of the Parallel2 model, the discount parameter is difficult to interpret for reasons discussed above, while in the Mixed model discounting occurs at two different “levels.” We therefore fit the Parallel model to each spacing condition individually and found the average best-fitting discount factor. Our results are summarized in Table 3.

**Table 3:** **Summary statistics of the best-fitting discount parameters for Parallel model in each condition**.

**Condition**	**Mean discount parameter**	**Standard deviation of discount parameters across subjects**
$3000 single reward	0.135	0.484
Three $1000 rewards with 12 month spacing	0.262	0.728
Three $1000 rewards with 60 month spacing	0.189	0.671
$1000 single reward	0.092	0.244

Surprisingly, the mean discount parameter for the $3000 reward case was slightly larger than for the $1000 reward case. However, matched-samples pairwise comparisons (*df* = 24), demonstrated that none of the differences in discount parameters across conditions were significant (Table 4).

**Table 4:** **Pairwise comparisons between the best-fitting discount parameters for the Parallel model in each condition**.

	**$3000 single**	**12 mo. spacing**	**60 mo. spacing**	**$1000 single**
$3000 single				
12 mo. spacing	*t* = −1.539, *p* = 0.14			
60 mo. spacing	*t* = −1.31, *p* = 0.20	*t* = 0.77, *p* = 0.45		
$1000 single	*t* = 0.87, *p* = 0.40	*t* = 1.67, *p* = 0.11	*t* = 1.09, *p* = 0.29	

## Model recovery

The next question we addressed is whether our data were sufficiently powered to discriminate between the models. The BICs for the models are quite high, reflecting the generally high level of variance we observed in responses. The average unsigned residual error across participants and across models was $272.19. Since the difference in BIC between models is rather low, one concern is whether our model comparison procedure is actually capable of picking up on the relatively subtle differences between the models. To address this, we performed a model recovery analysis in which we created model-based data with a level of noise comparable to that actually observed. We then examined whether the model used to generate the data offered a significantly better fit than the alternative model. Because our primary aim in this study was a comparison between the HDTD and Parallel models, we limited the model recovery to these two models. For each participant, we generated two sets of simulated data, one for each model, using the best-fitting discount parameters for the model in question. We then added Gaussian noise with a mean of 0 and a standard deviation that matched the standard deviation of the residual errors for the particular participant and particular model. In this way, we created simulated data with the same parameters and noise as the human subject data, but with a known underlying generative model.

For each set of simulated data, we fit each of the two models, calculated the corresponding BIC values, and conducted a matched-samples *t*-test just as we did in the analysis of the genuine data. The *t*-tests were one-tailed since we were testing only whether the data-generating model offered a better fit, and Bonferonni-corrected with an alpha of 0.025. We found that in both cases, the correct model offered a significantly better fit. For the HDTD-generated data, the HDTD model had an average BIC of 324.51 while the Parallel model had an average BIC of 325.53. The HDTD model offered a better fit with *p* = 0.0053. For the Parallel-generated data, the HDTD model had an average BIC of 318.21 while the Parallel model had an average BIC of 317.05. The Parallel model provided the better fit with *p* = 0.0035. These findings demonstrate that despite the high amount of noise and relatively similar performance of the models, our model discrimination procedure is able to determine the better fitting model for the observed data.

## Discussion

In this study we fit several models of temporal discounting to behavioral data gathered from a procedure which estimated the value placed on various hypothetical monetary rewards and reward sequences. Of particular interest were the Parallel and HDTD models. We found that the Parallel model performed most similarly to human subjects while the HDTD model performed relatively poorly. Our findings are consistent with the hypothesis that, within sequences of rewards, individual rewards are discounted independently. It is notable that in our analyses, all discounting models that assumed at least some form of independent discounting of rewards (Parallel, Exponential, Mixed model) were ranked higher than models that assumed some interaction amongst rewards (HDTD, Serial). Although the Mixed model treats sequences of rewards as a “package” reward that is then discounted, individual rewards within a sequence are discounted independently in order to determine the value of the package at the beginning of the reward sequence.

The finding that models that discount rewards independently provide better fits to our data is consistent with the findings of Brunner and Gibbon (1995). In their design, rats chose between a spaced sequence of rewards or a massed option in which the rewards were delivered one immediately after the other. Rather than varying the magnitude of the massed option to estimate the value of the spaced option, as we did here, they varied the time delay to the massed option. In contrast to our results however, they found that the Mixed model did a poor job of accounting for the rats' behavior. In accord with our findings, though, Mitchell and Rosenthal (2003) found that both the Parallel model and the Mixed model accurately characterized temporal discounting in rats. The Parallel model was also found by Kirby (2006) to accurately predict the behavior of human subjects making choices about real monetary rewards.

As can be seen in Tables 3, 4 we failed to replicate the magnitude effect, which occurs when larger rewards are discounted less heavily than smaller rewards, despite the fact that it has been found in a wide variety of other discounting studies (e.g., Myerson and Green, 1995; Kirby, 1997; Johnson and Bickel, 2002). In particular, the average discount rate in the small single reward condition was actually slightly lower than the average discount rate in the large single reward condition, though not significantly so. One possible reason for this is that the two reward values, $1000 and $3000 were not different enough to elicit the magnitude effect. In Myerson and Green (1995), for instance, the rewards differed by a factor of 10 as the small reward was $1000 and the large reward was $10,000.

Recall that both the HDTD model and Kurth-Nelson and Redish's (2009) μAgents model are capable of implementing hyperbolic discounting in the context of temporal difference learning, which has traditionally incorporated the less behaviorally plausible exponential discounting. A primary motivation for this study was to test which method was favored by behavioral data. Our results provide support for the hypothesis that individual rewards within a sequence are discounted independently from one another. Although these results are consistent with the μAgents model, it remains an open question as to whether and where hypothesized distributions of exponentially discounted representations of reward are maintained and integrated in the brain. Presumably, in order for an agent to exhibit hyperbolic discounting at the behavioral level, the distributed representations suggested by the μAgents model would need to be integrated prior to the generation of a response. While regions within the brain have been observed whose activity is consistent with distributed discount factors (Tanaka et al., 2004), it has not been established that such signals represent exponential rather than hyperbolic discount parameters. Practically, distributed representations of hyperbolic discount parameters would manifest behaviorally in much the same way that distributed representations of exponential discount parameters do (i.e., as a hyperbolic discount function) for much the same reason that hyperbolic discounting is observed both at the level of individual subjects and at the group level (e.g., Green and Myerson, 1996). Moreover, activity in regions within the brain implicated in discounting and reward processing tends to be more consistent with hyperbolic rather than exponential discounting (Paulus and Frank, 2006; Kobayashi and Schultz, 2008). Future work is needed to determine how putative exponentially discounted reward representations are integrated in order to yield hyperbolic discounting.

### Conflict of interest statement

The authors declare that the research was conducted in the absence of any commercial or financial relationships that could be construed as a potential conflict of interest.
